# Reporting of National Cancer Registry statistics in Europe: a scoping review

**DOI:** 10.1016/j.esmorw.2026.100752

**Published:** 2026-09-17

**Authors:** B.P. Pang, K.C.W.J. Ebben, I. Cara, A. Duratović Konjević, G. Geleijnse

**Affiliations:** 1 Department of Research and Development, Netherlands Comprehensive Cancer Organisation (IKNL), Utrecht, The Netherlands; 2 Department of Epidemiology, GROW-School for Oncology and Developmental Biology, Maastricht University, Maastricht, The Netherlands; 3 Slovenian Cancer Registry, Institute of Oncology Ljubljana, Ljubljana, Slovenia; 4 Cancer Registry of Norway, Norwegian Institute of Public Health, Oslo, Norway

**Keywords:** cancer registry, cancer statistics, data communication, Europe, timeliness

## Abstract

**Background:**

Population-based cancer registries (PBCRs) play an important role in national cancer control programs by providing data for cancer statistics. The European Network of Cancer Registries (ENCR) aims to promote the impact and use of European PBCRs and umbrella organizations (UOs). This study maps the reporting practices of European national PBCRs (NPBCRs) and UOs regarding the key statistical indicators: incidence, prevalence, and survival.

**Patients and methods:**

The ENCR Members Contact List and Google.com were used to identify websites of all NPBCRs and UOs, which were screened for sources of evidence (SoEs). Using a predefined protocol, the SoEs were assessed for the reporting of incidence, prevalence, and survival, as well as the timeliness of reporting. A scoring rubric was used to assess data accessibility.

**Results:**

Excluding microstates, at least one SoE was identified for 34 out of 45 European countries. In total, 60 SoEs containing nationwide statistics were found, mainly general cancer reports (*n* = 23) and dashboard tools (*n* = 20). Nationwide cancer incidence was reported in all 34 countries, whereas prevalence and survival were reported in approximately half of the SoEs. The time lag between publication year and most recently covered year ranged from 0 to 10 years (mean 2.69 years). The average accessibility score of SoEs was 7 out of 10 points.

**Conclusion:**

This study highlights the heterogeneity in the reporting, accessibility, and timeliness of cancer statistics across European NPBCRs and UOs. Improving harmonization and reporting practices could enhance the value of cancer registry data for cancer control.

## Highlights

• Cancer incidence was reported by all included European registries.
• Survival and prevalence data were reported by approximately half of the registries.
• Reporting lag ranged from 0 to 10 years (mean 2.7 years).
• Accessibility of European cancer registry data was assessed.
• Improving timeliness and accessibility may strengthen registry impact.

## Introduction

Population-based cancer registries (PBCRs) play a crucial role in national cancer control programs because they aim to provide accurate, comprehensive data on the cancer burden of a region.^1^ Cancer burden is commonly quantified using key statistical indicators, such as incidence, prevalence, and survival. Incidence measures the number of new cancer cases within a specified period, prevalence reflects the number of individuals living with a previous cancer diagnosis, and survival describes the proportion of patients alive following diagnosis.^2^

Among European PBCRs, heterogeneity exists in reporting practices, size of population coverage, and resource capacity.^3^^,^^4^ Most countries, such as Croatia and Norway, have a single national PBCR (NPBCR) that covers nearly the entire population. Other countries, such as Germany, achieve nearly full coverage with multiple regional PBCRs, coordinated by an umbrella organization (UO). A third group of countries, such as Spain, has only partial population coverage through a UO that coordinates with available regional PBCRs. However, not all European countries are covered by a functional NPBCR or UO. For these countries, data from comparable countries are used to estimate the cancer burden.^5^^,^^6^

The European Network of Cancer Registries (ENCR) aims to promote the harmonization and comparability of cancer registry data across Europe through the standardization of registration practices and data collection.^5^ Despite these efforts, considerable variation remains in the quality, timeliness, and accessibility of the data reported by European PBCRs.^4^^,^^7^ These characteristics are essential for generating reliable evidence to support cancer surveillance, research, and cancer control planning.^8^ To facilitate comparisons of cancer burden across Europe, the European Cancer Information System (ECIS) provides statistics based on the data contributed by PBCRs within the ENCR, enabling the exploration of geographical patterns and temporal trends in incidence, prevalence, and survival.^9^

A better understanding of how European NPBCRs and UOs report their national statistical indicators is essential to assess their potential to monitor and impact national cancer control programs.^10^ Although the ECIS supports comparison between European countries, its reporting may not always provide the detail and interpretation required to inform national cancer control efforts. In this context, clear communication of the cancer burden to relevant stakeholders, including the public, policymakers, and health care professionals, is critical.^10^^,^^11^ It enables evidence-based and targeted prevention, planning, and resource allocation, as well as early detection and development of treatment.^1^^,^^12^

To the best of our knowledge, no research has yet examined how European countries report the key statistical indicators. Therefore, this scoping review aims to map the current reporting practices of incidence, prevalence, and survival, including the timeliness and accessibility of reported data. A better understanding of these reporting practices among European NPBCRs and UOs will help improve harmonization of the reported statistical indicators, foster stronger collaboration between European cancer registries, and potentially identify best practices. Ultimately, these improvements may contribute to more effective national cancer control plans and a reduced burden of cancer.

### Rationale and objectives

Scoping reviews aim to systematically identify and map all the evidence on a particular topic, often irrespective of the source.^13^ Due to the multidimensional research question and the inclusion of diverse publication types in European NPBCRs and UOs, the scoping review approach was considered the most suitable methodological approach.^14^^,^^15^

The goal of this study was to systematically map the current reporting practices of European NPBCRs and UOs. To achieve this, the main objective was to analyze how incidence, prevalence, and survival are reported on a national level for the European countries. In addition, the reporting of these indicators was assessed for timeliness and accessibility for potential users.

## Methods

### Framework

This study followed the scoping review framework developed by the Joanna Briggs Institute.^13^^,^^16^ The study was conducted and reported in accordance with the Preferred Reporting Items for Systematic Reviews and Meta-Analyses (PRISMA) extension for scoping reviews.^14^ A protocol was developed collaboratively a priori before conducting the study (Supplementary Appendix S1, available at https://doi.org/10.1016/j.esmorw.2026.100752). This protocol was not published in advance.

### Identification of websites of European NPBCRs and UOs

The website of each NPBCR or UO responsible for nationwide cancer statistics was identified for eligible countries. Eligible countries included Cyprus and all European countries,^17^ except Russia and Belarus, which were excluded for geopolitical reasons, and European microstates (Andorra, Liechtenstein, Monaco, San Marino, and Vatican City), which were excluded because of their limited population size. The UK was considered to represent four separate countries (England, Scotland, Wales, and Northern Ireland).

The Members Contact List of the ENCR was used as the primary source for website identification.^5^ The following filters were used: ‘All sites registries’ and ‘All ages registries’ with ‘Full members’ or ‘Associate members’. If the website link was unavailable or faulty, an internet search using Google was conducted using the following queries:

• For NPBCRs: ‘[country name] cancer registry’.
• For UOs: ‘[country name]’s network of cancer registries’.

Countries for which no NPBCR or UO website could be identified after applying the search strategy were excluded from further analyses. Although Gibraltar was on the Members Contact List, it was excluded because it is not a country.

The search strategy was carried out by reviewer 1 (BPP) as the primary reviewer and reviewers 2-5 (KCWJE, GG, ADK, and IC), each covering one quarter of the European countries, as secondary reviewers. Any discrepancies were resolved through discussion between the two reviewers involved. The search and subsequent analyses were conducted from 1 December 2025 to 28 January 2026.

### Identification and selection of sources of evidence

The identified websites were screened for sources of evidence (SoEs) using the following key words and their synonyms: ‘(Cancer) Registries’, ‘Publications’, ‘Documents’, ‘Statistics’, ‘Tool(s)’, and ‘Data’. The search queries and key words were applied in both English and, where necessary, the native language using Google Translate. Several types of SoEs were considered eligible for identification:

General cancer reports—reports that provide an overview of the national cancer burden—typically include multiple statistical indicators such as incidence, prevalence, and survival.

Indicator-specific cancer reports are similar to general cancer reports but focus primarily on a single statistical indicator, such as incidence, prevalence, or survival.

Documents containing tables only are the documents presenting cancer burden statistics predominantly in tabular format, with limited or no accompanying narrative text or interpretation.

Dashboard tools are interactive web-based platforms that allow users to generate, explore, and visualize cancer burden statistics through customized tables, graphs, or other data displays.

Inclusion criteria for SoEs were reporting of at least one statistical indicator (incidence, prevalence, and/or survival), public accessibility from an eligible website, coverage of all age groups, and all cancer sites (with or without coverage of nonmelanoma skin cancer). SoEs were excluded if they were specific to a single cancer site or if they covered data from childhood-only cancer registries and/or hospital-based cancer registries.

As multiple eligible SoEs were often available for a single NPBCR or UO, a predefined selection hierarchy was applied to ensure a consistent and manageable approach to data extraction. Dashboard tools were included as SoEs whenever available. In addition to dashboard tools, the most comprehensive type of SoE was selected according to the predefined hierarchy: firstly, general cancer reports; secondly, factsheets on cancer burden; and thirdly, statistical documents containing tables only.

The identification and selection of SoEs were conducted using the same reviewer allocation and consensus procedure as described for website identification.

### Data charting process and data items

Data extraction was conducted using separate Excel tables for website identification, SoE identification, and the core statistical indicators incidence, prevalence, and survival. The complete data extraction item sets are provided in the Supplementary Appendix S2 (available at https://doi.org/10.1016/j.esmorw.2026.100752). The item lists for each table are as follows:

• Website identification: ‘Country, PBCR name, website link, location website link (ENCR website or Google.com)’.
• SoE identification: ‘Country, SoE type(s), name SoE(s), website link of SoE’.
• Incidence: ‘Country, name SoE(s), reported Y/N, trend prediction Y/N, stratification by gender, age, cancer site, stage at diagnosis, geographical region Y/N’.
• Prevalence: ‘Country, name SoE(s), reported Y/N, stratification by gender, age, cancer site, stage at diagnosis, geographical region Y/N, reported timeframe(s) in years’.
• Survival: ‘Country, name SoE(s), reported Y/N, stratification by gender, age, cancer site, stage at diagnosis, geographical region Y/N, reported timeframe(s) in years’.
• Timeliness: ‘Most recent year of coverage’, ‘year of publication/last update’.

In addition, the accessibility of each SoE was evaluated. A predefined scoring rubric was designed to assess the accessibility of data from the perspective of potential users. Five criteria were scored from 0 to 2 points, allowing a total score to summarize the overall accessibility of each SoE: ‘easy to find (number of clicks from homepage)’, ‘easily accessible/viewable’, ‘use of graphs/visualizations’, ‘main findings summarized’, and ‘glossary/guidance for interpretation’. This scoring rubric was a novel methodological approach because no existing validated assessment tool could be identified.

## Results

### Website identification

**Figure 1:**
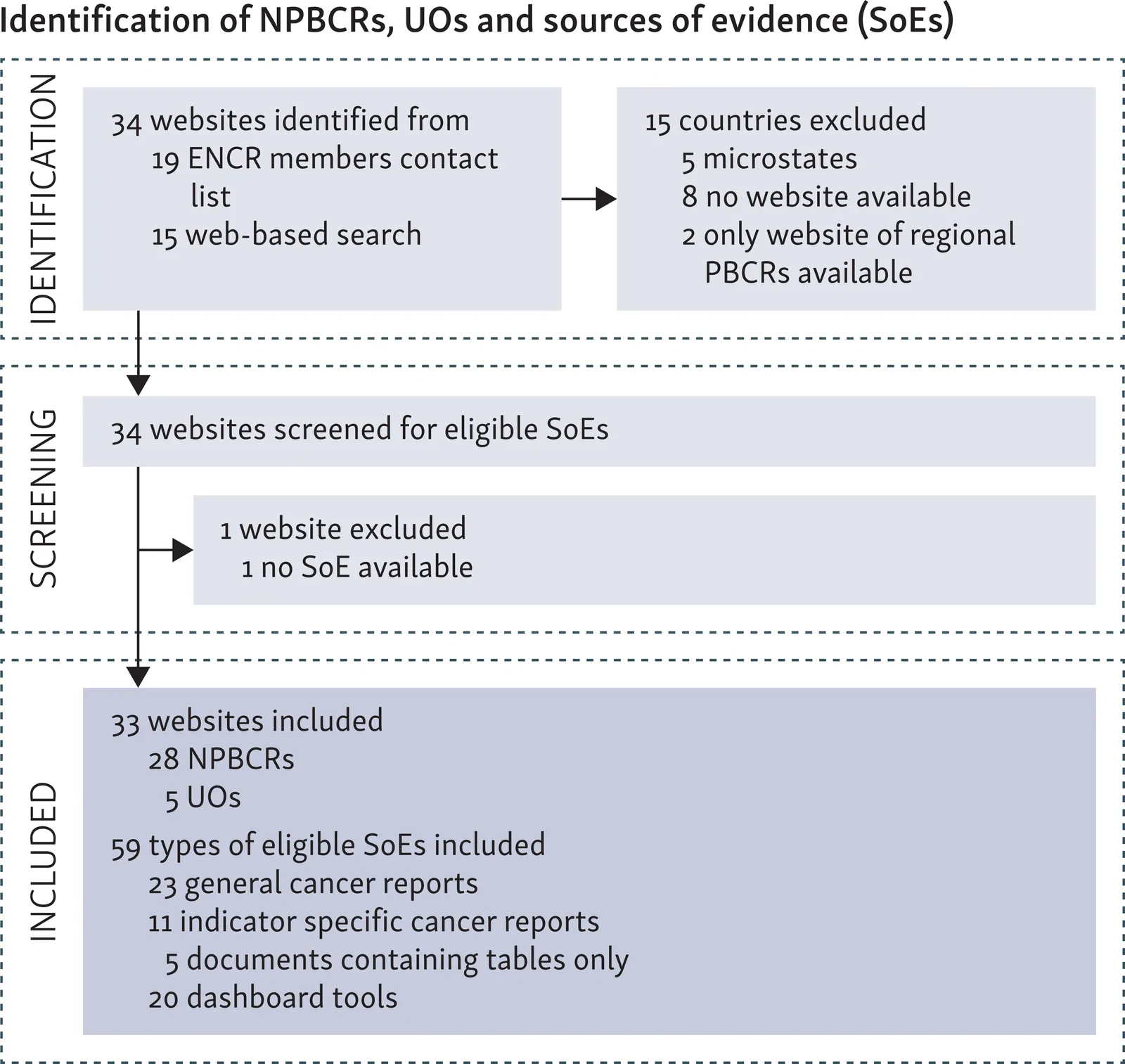
**Preferred Reporting Items for Systematic Reviews and Meta-analyses (PRISMA)2020–based flow diagram****^18^****showing the identification of websites of NPBCRs and UOs, as well as the number of SoEs eligible for data extraction.** ENCR, European Network of Cancer Registries; NPBCR, National Population-Based Cancer Registry; PBCR, Population-Based Cancer Registry; SoE, sources of evidence; UO, umbrella organization.

Forty countries were identified as active members of the ENCR. A website of an NPBCR or UO could be identified for 34 of these 40 countries, via the ENCR Members Contact List (*n* = 19) and via the Google search (*n* = 15). Four countries (Bosnia and Herzegovina, Moldova, Montenegro, and Turkey) were excluded because no website could be identified using the predefined search strategy. Excluding microstates, four European countries were not the members of the ENCR (Albania, Kosovo, North Macedonia, and Greece), but no website could be found via the Google search either. Two countries (Portugal and Romania) were excluded because only websites of the regional cancer registry were available (Figure 1).^18^

### SoEs identification

All 34 websites of the NPBCRs and UOs were screened, and at least one SoE was identified from 33 websites, of which 28 were from NPBCRs and 5 were from UOs (France, Germany, Italy, Spain, and Switzerland). Out of the 33 websites, a total of 59 SoEs were identified: 16 contained one SoE, 11 contained two SoEs, and 6 contained three or more SoEs (Table 1). Different types of SoEs were classified according to a predefined hierarchy: general cancer reports were the most common type of SoEs (*n* = 23), followed by dashboard tools (*n* = 20) and indicator-specific cancer reports (*n* = 11) (Figure 1). Currently, only 20 out of 33 included NPBCRs and UOs have a dashboard tool available (Table 1).

**Table 1:** Overview of included countries and their respective SoEs

Country	Name of SoE	SoE type	Incidence (out of 6 items)	Prevalence (out of 5 items)	Survival (out of 5 items)	Time lag	Accessibility score
Austria	Krebserkrankungen in Österreich 2025	Annual cancer report	5	2	4	2	9
Belgium	Cancer burden in Belgium 2004-2017	General cancer report	5	3	4	3	8
	Tekenmodule	Dashboard tool	5	NR	NR	1	6
Bulgaria	Регистрирани заболявания и умирания от злокачествени новообразувания в Република България, 2025 г	Annual cancer report	3	NR	NR	1	6
Croatia	Incidencija raka u Hrvatskoj u 2023. godini	Document containing tables only	4	NR	NR	2	5
Cyprus	Cyprus Cancer Registry Progress Report, August 2016	General cancer report	3	NR	NR	3	4
Czech Republic	Cancer incidence 2019-2021 in the Czech Republic	General cancer report	5	2	3	2	10
Denmark	Kræftoverlevelse i Danmark 2009-2023	Indicator-specific cancer report	NR	NR	2	2	8
	Nye kræfttilfælde i Danmark 2024	Indicator-specific cancer report	4	3	NR	1	6
Estonia	Cancer in Estonia: incidence 2022, survival 2018-2022, and HPV-related cancers 1998-2022	General cancer report	4	2	3	3	10
Finland	Cancer in Finland 2023	Annual cancer report	4	2	2	2	10
	(No name) ‘Interactive statistic application’	Dashboard tool	4	3	3	2	6
France	Panorama des cancres en France (Edition 2023)	General cancer report	2	1	NR	0	8
	Survie des personnes atteintes de cancer en France métropolitaine 1989-2018	General cancer report	NR	NR	3	3	6
Germany	Krebs in Deutschland für 2021-2023	Annual cancer report	5	2	3	2	10
	Datenbankabfrage	Dashboard tool	3	3	3	1	4
Hungary	A rosszindulatú daganatos betegségek incidenciájának és mortalitásának vármegyénkénti különbségei Magyarországon 2005 és 2019 között	Indicator-specific cancer report	2	NR	NR	3	6
	Comparison of Cancer Survival Trends in Hungary in the Periods 2001-2005 and 2011-2015 According to a Population-Based Cancer Registry	Indicator-specific cancer report	2	NR	2	7	8
	No name	Dashboard tool	4	NR	NR	3	2
Iceland	Tölulegar upplýsingar	Dashboard tool	5	2	2	1	8
Ireland	Cancer in Ireland 1994-2022	General cancer report	3	2	2	2	8
	Incidence statistics	Dashboard tool	4	NR	NR	3	8
	Survival statistics	Dashboard tool	NR	NR	5	3	8
Italy	I NUMERI DEL CANCRO IN ITALIA 2023	Annual cancer report	3	1	2	1	8
Latvia	Health Statistics Database—malignant neoplasms	Dashboard tool	5	3	2	1	4
Lithuania	Vėžys Lietuvoje 2017 metais	Annual cancer report	4	NR	2	6	5
	Vėžio registro informacinė sistema	Dashboard tool	5	3	5	2	8
Luxembourg	Le cancer au Luxembourg: Incidence et mortalité 2014 - 2021- Rapport du Registre National du Cancer du Luxembourg: 1re édition Rapport du Registre National du Cancer du Luxembourg	General cancer report	3	NR	NR	4	8
Malta	All Malignant Cancers	Document containing tables only	2	NR	NR	4	4
Norway	Cancer in Norway 2024	Annual cancer report	5	3	4	1	9
	Statistikkbank Kreftregisteret	Dashboard tool	4	4	NR	1	8
Poland	Reports online'	Dashboard tool	5	3	3	NR	8
	Nowotwory złośliwe w Polsce w 2022 roku	Annual cancer report	4	NR	NR	2	9
Serbia	MALIGNANT TUMOURS IN REPUBLIC OF SERBIA 2024	Annual cancer report	4	NR	NR	2	6
Slovakia	Interaktívna prezentácia výstupov Národný onkologický register Slovenskej republiky (NOR SR)	Dashboard tool	5	NR	5	10	7
Slovenia	Cancer in Slovenia 2022	Annual cancer report	4	2	2	3	10
	SLORA	Dashboard tool	5	2	2	3	8
Spain	Estimates of incidence in Spain 2025	Indicator-specific cancer report	3	NR	NR	0	8
	Cancer prevalence in Spain as of 31 December 2020	Indicator-specific cancer report	NR	2	NR	5	10
	Supervivencia de Cáncer en Espańa, 2002-2013	Indicator-specific cancer report	NR	NR	4	7	7
Sweden	Statistikdatabas för cancer	Dashboard tool	5	NR	NR	1	6
	Cancer i Sverige 2025—Insjuknande och dödlighet	Indicator-specific cancer report	5	NR	NR	2	6
	Cancer i Sverige 2025–Relativ överlevnad 1974-2023	Indicator-specific cancer report	NR	NR	5	2	6
Switzerland	Krebsinzidenz 1983-2022	Document containing tables only	4	NR	NR	3	2
	Krebsprävalenz 2016-2026	Document containing tables only	NR	3	NR	3	3
	Krebsüberleben 1980-2022	Document containing tables only	NR	NR	2	3	2
	Krebsmonitoring Schweiz	Dashboard tool	4	NR	NR	3	4
	MonAM	Dashboard tool	4	3	NR	4	8
The Netherlands	Kanker in Nederland: prognoses tot en met 2032	Annual cancer report	4	3	3	3	10
	NKR Cijfers	Dashboard tool	5	5	5	1	6
Ukraine	РАК В УКРАÏНІ, 2022-2023	Annual cancer report	5	2	NR	2	10
England	Cancer incidence and mortality dashboard	Dashboard tool	5	NR	NR	2	6
	Cancer Registration Statistics, England, 2023	General cancer report	4	NR	NR	2	9
	Cancer survival dashboard	Dashboard tool	NR	NR	5	5	6
	Cancer prevalence dashboard	Dashboard tool	NR	4	NR	3	6
Scotland	Cancer incidence and prevalence in Scotland	General cancer report	4	NR	NR	2	8
Northern Ireland	All cancers 1993-2022	General cancer report	3	2	NR	3	7
Wales	Cancer incidence in Wales	Indicator-specific cancer report	5	NR	NR	3	6
	Cancer survival in Wales	Indicator-specific cancer report	NR	NR	5	4	6
Total			4.04/6	2.57/5	3.23/5	2.67 years	6.92 points

This table presents all countries with an available website of a National Population-Based Cancer Registry or umbrella organization, along with the identified SoEs. For each SoE, the reporting of incidence, prevalence, and/or survival is indicated, including the number of stratification variables. The time lag (in years) is also shown, defined as the difference between the year of publication and the most recent year of data covered in the SoE. The final column displays the accessibility score assigned to each SoE according to the scoring rubric.

HPV, human papillomavirus; NR, not reported; SoE, source of evidence.

### Statistical indicators

All 59 SoEs reported at least one of the key statistical indicators. Incidence was reported most frequently. Across Europe, on an average, four out of six items were reported for incidence, and for prevalence and survival, the average was 2.6 out of 5 and 3.2 out of 5 items, respectively (Table 1). Timeliness of data was assessed on the basis of the lag between the published year or last updated year for dashboard tools and the last year covered in the report, ranging from 0 to 10 years, with an average of 2.7 years. However, it is important to note that this range covers both observed and predicted cancer statistics, without differentiation in this study. Country-specific results for incidence, prevalence, survival, and timeliness are presented in Supplementary Tables S1 and S2 (available at https://doi.org/10.1016/j.esmorw.2026.100752).

### Overall reporting of incidence, prevalence, and survival

All included NPBCRs and UOs reported incidence in at least one SoE. Of the 59 SoEs, 11 did not report incidence; however, these were all indicator-specific dashboard tools or cancer reports focusing exclusively on prevalence or survival. Overall, incidence reporting was frequently stratified by sex, age, and cancer type. Stage at diagnosis and geography were less frequently stratified, whereas only 7 of 48 SoEs reported future trend predictions of incidence (Table 2).

**Table 2:** Overview of the number of SoEs that stratified incidence, prevalence, and survival, by each included item, and the number of countries that stratified the item in at least one SoE

Items	Incidence			Prevalence			Survival		
	Yes	No	Countries	Yes	No	Countries	Yes	No	Countries
Sex	46	2	33	25	4	19	27	3	22
Age	41	7	31	7	22	5	15	15	14
Cancer type	47	1	32	28	1	22	29	1	23
Stage at diagnosis	22	26	18	3	26	2	14	16	14
Geography	31	17	11	9	20	8	12	18	11
Future trend	7	41	6	NA	NA	NA	NA	NA	NA

NA, not applicable; SoE, source of evidence.

Out of 33 countries, prevalence was reported in at least one SoE by 23 countries. Approximately half of the SoEs (29 out of 59) reported prevalence. Nine SoEs were indicator-specific cancer reports focusing on incidence and/or survival. Prevalence was commonly stratified by sex and cancer type, whereas age, geography, and stage at diagnosis were less frequently stratified (Table 2).

Twenty-three out of 33 countries reported survival in at least one SoE. Thirty SoEs reporting survival were identified. Survival was stratified by sex and cancer type for nearly all SoEs, whereas stratification by age, stage at diagnosis, and geography was less common (Table 2).

### Accessibility

On an average, the SoEs scored ∼6.9 out of 10 points. Most SoEs (*n* = 52) scored well for the criterion ‘easy to use/view’, making it the strongest aspect overall. The majority of the SoEs provided a summary of their main findings (*n* = 37). In terms of access, just over half of the SoEs (*n* = 33) could be found within three clicks from the homepage, whereas 21 SoEs required more than five clicks. Graphs or visualizations were also provided in the majority of the SoEs (*n* = 46). In more than half of the SoEs (*n* = 38), a glossary or guide for interpretation was available (Table 1).

Dashboard tools included more graphs compared with other types of SoEs. On the contrary, documents containing tables only did not include graphs, summaries, or glossaries. More often than not, general cancer reports accompanied the data with graphs, glossaries, and summaries (Table 1; Supplementary Table S3, available at https://doi.org/10.1016/j.esmorw.2026.100752).

## Discussion

Presenting national-level population-based cancer statistics is a key mechanism for NPBCRs and UOs to impact national cancer control programs. Accessible, fit-for-purpose reports and dashboards are therefore important mechanisms to inform about the state-of-play of cancer. Established principles about data quality in cancer registries already exist.^8^^,^^19^ This study focuses mainly on the principles of ‘comparability of statistics’ and ‘timeliness of reporting’ by European cancer registries. Additionally, the concept of ‘accessibility of cancer registry statistics’ was introduced in this study.

This study mapped how European countries report their nationwide statistical indicators, as well as how timely and accessible they are. For 10 countries in Europe (including three European Union member states, excluding microstates), no nationwide population-based cancer statistics could be identified. The lack of these statistics may hinder the development of cancer control strategies, such as national cancer control programs. In this context, the ENCR could play an important role in improving coverage, comparability, and completeness of data reporting by European cancer registries.

Heterogeneity was found regarding the depth of reporting incidence, prevalence, and survival. Although incidence was reported by all included NPBCRs and UOs, stratification by geography, stage at diagnosis, and future trends was often limited. Additionally, predictions for future cancer incidence were reported by only six countries. However, standardized prediction models for NPBCRs and UOs such as the NordPred model^20^^,^^21^ could be used without the need for additional data collection.

The reporting of prevalence and survival was less consistent, and both indicators were less frequently stratified compared with incidence. These findings point to potential areas for improvement given the need for governments to recognize NPBCRs and UOs as key policy tools.^3^ Insufficient stratification of prevalence and survival reduces their utility. However, differences in resources, data infrastructure, or registry maturity may explain why not all European countries are capable of reporting all statistical indicators. Although the ECIS provides comparable cancer burden indicators across Europe, the present study demonstrates that inconsistencies in the reporting of statistical indicators may reduce the value of ECIS.

Timeliness as a principle of data quality in cancer registries was first introduced by Bray and Parkin.^8^ Although there is no formal definition, timeliness relates to the speed with which a registry is able to collect, process, and report reliable cancer data. Therefore, timely reporting by cancer registries requires adequate personnel and efficient data transfer infrastructure.^8^ Delays in reporting may limit the relevance of data, making it less useful for timely decision making. To date, guidelines about timeliness are still missing, but initiatives such as CancerWatch aim to improve data quality and timeliness across cancer registries in Europe.^22^

Visual communication of health data, such as graphs, charts, and diagrams, is essential for accessible cancer statistics.^23^ Many SoEs have provided executive summaries that highlight advances and challenges in cancer control. Clear presentation of the statistics enhances usability and impact of all PBCRs while also strengthening the communication with key stakeholders, including health care professionals, policy makers, researchers, media, and the general public.^11^^,^^24^ Dashboard tools have great potential to increase the exploration and dissemination of cancer registry data.^11^ This study found that fewer than half of the countries have dashboard tools available. Open-source dashboard and visualization tools are available and may benefit resource-constrained PBCRs.^25^

This scoping review has some limitations. Some SoEs may not have been identified via the study protocol. A predefined hierarchy was used to determine the inclusion of SoEs, aiming to include the most comprehensive sources. However, some PBCRs may have more detailed documents that contain tables only, compared with their general cancer reports. Therefore, loss of information may have happened during data extraction. The predesigned item sets for data extraction included the most important aspects regarding statistical indicators and accessibility. However, they may not fully capture stratification practices, such as socioeconomic status or treatment by cancer type. Additionally, this study did not distinguish between observed and predicted cancer statistics. Some countries predicted statistics on the basis of existing data when full national coverage is not available. To understand the timeliness of data reported by cancer registries, further work is required.

This study did not examine the principles of ‘validity’ and ‘completeness’ of cancer registry data.^8^^,^^19^ Additionally, cancer mortality was not examined in this study because the focus was on data originating from PBCRs. Mortality statistics in Europe are often registered by an organization different from cancer registries.^26^ Future research evaluating these fields would further strengthen the understanding of the cancer burden across Europe. Regarding the harmonization efforts by the ECIS and ENCR to improve the comparability of European data, there is an opportunity to standardize the underlying statistical data analysis and generation of tables and graphs.

In conclusion, this scoping review provides insight into current reporting practices of NPBCRs and UOs across Europe and highlights substantial heterogeneity in the reporting, accessibility, and timeliness of cancer statistics. Improving harmonization and reporting practices could enhance the value of cancer registry data for cancer control. As the cancer burden continues to increase, the need for timely, accessible, and comprehensive cancer statistics to inform cancer control will become even more critical.

## Declaration of AI and AI-Assisted Technologies in the Writing Process

During the preparation of this work, the authors used ChatGPT in order to enhance clarity and check the grammar and spelling. After using this tool/service, the authors reviewed and edited the content as needed and take full responsibility for the content of the published article.
