# Supplementary material for: Reporting of National Cancer Registry statistics in Europe: a scoping review
Source: ESMO Real World Data Digit Oncol. 2026 Sep 17;14:100752. doi: 10.1016/j.esmorw.2026.100752 (PMC13602766; doi:10.1016/j.esmorw.2026.100752)
Supplement: Supplementary file 1 [file mmc1.docx]

Protocol for the scoping review ‘Reporting of National Cancer Registry Statistics in Europe’

Bob P. Pang^1,4^ Kees C.W.J. Ebben^1,3^, Irene Cara^1^, Amela Duratović Konjević^2^, Gijs Geleijnse^1,5
1^Netherlands Comprehensive Cancer Organisation (IKNL), Rijnkade 5, 3511CV Utrecht, The Netherlands
^2^Slovenian Cancer Registry, Institute of Oncology Ljubljana, Zaloška cesta 2, 1000 Ljubljana, Slovenia
^3^Department of Epidemiology, GROW-School for Oncology and Developmental Biology, Maastricht University, P. Debyeplein 1, 6229HA Maastricht, The Netherlands
^4^Present address: Erasmus MC, Dr. Molewaterplein 40, 3015GD Rotterdam, The Netherlands
5Present address: Norwegian Institute of Public Health, [Ullernchausséen 64, 0379 Oslo,](https://www.bing.com/ck/a?!&&p=e7b74df143b086ab0fce6bd1738828d1a5b9b72e5dccd5744f95c5f880a87e4dJmltdHM9MTc3NjI5NzYwMA&ptn=3&ver=2&hsh=4&fclid=1f0c7941-d77a-6671-0574-6f33d6616735&u=a1L21hcHM_Jm1lcGk9MH5-RW1iZWRkZWR-QWRkcmVzc19MaW5rJnR5PTE4JnE9Q2FuY2VyJTIwUmVnaXN0cnklMjBOb3J3YXkmc3M9eXBpZC5ZTkY2N0RCQjdDQTY5MUMxQjYmcHBvaXM9NTkuOTMwODkyOTQ0MzM1OTRfMTAuNjY0Mjc3MDc2NzIxMTkxX0NhbmNlciUyMFJlZ2lzdHJ5JTIwTm9yd2F5X1lORjY3REJCN0NBNjkxQzFCNn4mY3A9NTkuOTMwODkzfjEwLjY2NDI3NyZ2PTImc1Y9MSZGT1JNPU1QU1JQTA&ntb=1) Norway
Corresponding author: Bob P. Pang, [bob.p.pang@gmail.com;b.pang@erasmusmc.nl](mailto:bob.p.pang@gmail.com;b.pang@erasmusmc.nl), Zaaierstraat 41, 2645JG Delfgauw, The Netherlands

## Background

In Europe, population-based cancer registries (PBCRs) collect data on cancer statistics that are essential for cancer control and research.^1^ The population coverage of PBCRs varies across Europe. Some countries, such as the Netherlands and Norway, have a single national PBCR (NPBCR) that covers the entire population. Others, such as Germany, achieve full coverage with multiple regional registries coordinating through an umbrella organization (UO). A third group of countries, such as Spain have only partial population coverage through either a single national cancer registry or an UO.^2,3^ One of the primary roles of a NPBCR is to measure the burden of cancer.^1^ This is achieved by collecting various statistics, including core statistical indicators such as incidence, prevalence, and survival. In addition, NPBCRs use these statistics to estimate future cancer trends, which is essential for facilitating cancer control programs.^1,4,5^

NPBCRs and UOs differ substantially in many aspects such as geographical coverage, size, reporting practices and funding.^6^ Therefore, understanding how each registry collects and presents data, is essential for improving harmonization and comparability. Collaboration between cancer registries through the European Network of Cancer Registries (ENCR) aims to improve the harmonization and comparability of data, as well as to facilitate the publication of European statistics via the European Cancer Information System (ECIS).^2^

Nevertheless, considerable variability exists in the quality, accessibility and timeliness of cancer registry data across Europe.^7^ The quality of these data depends on their fitness for purpose: they must provide a valid and trustworthy representation of cancer occurrence and outcomes in the population.^8,9^ Accessibility of cancer registry data is crucial for transparency and public engagement, but also for professionals who rely on the data for research. Timeliness—defined as the interval between diagnosis and the case being available in the registry^10^—is equally important, as rapid reporting benefits both health providers and researchers and can enhance the registry’s reputation.^8^

To the best of our knowledge, no research has examined how cancer registry data is reported by European NPBCRs and UOs. This study analyses how core statistical indicators–incidence, prevalence, survival–and their predicted trends are reported, as well as timeliness and accessibility of the reported data. A better understanding of the heterogeneity in cancer data reporting among European NPBCRs and UOs will help develop best practices, improve harmonization of statistical indicators and foster stronger collaboration between cancer registries across Europe.

## Rationale and Objectives

Scoping reviews aim to systematically identify and map all the evidence on a particular topic, often irrespective of the source.^11^ Due to the multi-dimensional research question and the inclusion of diverse publication types of European NPBCRs and UOs, the scoping review approach was considered the most suitable methodological approach.^12,13^

The goal of this study was to systematically map the current reporting practices of European NPBCRs and UOs. To achieve this, the main objectives were to analyze how incidence, prevalence and survival are reported on a national level for the European countries. In addition, the reporting of these indicators was assessed for timeliness and accessibility for potential users.

## Methods

#### Framework

This study follows the scoping review framework developed by the Joanna Briggs Institute.^11,14^ The study will be conducted and reported in accordance with the PRISMA extension for scoping reviews.^12^ This protocol includes the rationale, objectives and methodology of the study.

#### Eligibility Criteria

For each of the 38 member countries of the ENCR (excluding Russia and Belarus for geopolitical reasons), the website of the PBCR or UO responsible for nationwide cancer statistics will be identified. The country will be excluded from this study if there is no website available for a NPBCR or UO. Similarly, if no SoE is available on the identified website, the candidate will be excluded. SoEs will include different types of reporting by European PBCRs and UOs, such as general cancer reports, factsheets, dashboard tools and statistical documents containing tables only.

The SoE must be accessible to the public from the homepage of the website. Inclusion criteria are coverage of data for all age groups and all cancer sites, with or without skin cancer. Exclusion criteria are hospital-based and childhood-cancer only registries and cancer site–specific reports.

#### Information Sources

The Members Contact List of the ENCR and Google were used to identify the websites of the European PBCRs.^2^ The Google search engine was used only if the Members Contact List didn't provide a valid website link. Google Translate was used to screen websites and SoEs without an English version. The websites of the NPBCRs were used to locate SoEs.

#### Search Strategy

The websites of European PBCRs and relevant UOs will be identified primarily using the ENCR Members Contact List. The following filters will be used: ‘All sites registries’ and ‘All ages registries’ with ‘Full members’ or ‘Associate members’. If the website link is not available on this list, an internet search using Google will be conducted in English and the native language using the following queries:

- For national PBCRs: *“[country name] cancer registry”*.
- For UOs: *“[country name]’s network of cancer registries”*.

The identified websites will be screened for the following keywords: *“(Cancer) Registries”, “Publications”, “Documents”, “Statistics”, “Tool(s)” and “Data”,* in both English and the native language. The most recently published annual cancer reports, factsheets, dashboard tools and statistical documents will be considered as potential SoE. Publications must be freely available and accessible to the public.

#### Selection of Sources of Evidence

All SoE identified through the search strategy will be screened for eligibility. SoE will be included if they report at least one core statistical indicator (incidence, prevalence, or survival). If a PBCR or UO provides multiple types of SoE, a predefined hierarchy will be used to select the source(s) that will be included in this review. The order of preference is as follows: *first,* interactive dashboard tools (if available) AND *second,* general cancer reports OR *third,* factsheets on cancer burden OR *fourth,* statistical documents containing tables only. In case a PBCR or UO provides SoEs that are specific for an statistical indicator, a combination will be selected to cover incidence, prevalence and survival. The selection will be conducted by two independent reviewers, and any discrepancies will be resolved through discussion.

#### Data Charting Process and Data Items

Data extraction will be conducted using separate Excel tables for website identification, SoE identification, and the core statistical indicators incidence, prevalence, and survival. The item lists for each table are as follows:

- Website identification: *“Country, PBCR name, Website link, Location website link (ENCR website or Google.com)”*.
- SoE identification: *“Country, SoE type(s), Name SoE(s)”*.
- Incidence: *“Country, Name SoE(s), Reported Y/N, Trend prediction Y/N, Stratification by Gender, Age, Cancer site, Stage at diagnosis, Geographical region Y/N”.*
- Prevalence: *“Country, Name SoE(s), Reported Y/N, Stratification by Gender, Age, Cancer site, Stage at Diagnosis, Geographical region Y/N, Reported Timeframe(s) in Years”*.
- Survival: *“Country, Name SoE(s), Reported Y/N, Stratification by Gender, Age, Cancer site, Stage at diagnosis, Geographical region Y/N, Reported Timeframe(s) in Years”*.
- Timeliness: *“Most recent year of coverage”, “Year of Publication/Last Update”*.

#### Assessment of accessibility

In addition to the core statistical indicators (incidence, prevalence, survival), each selected SoE will also be assessed for accessibility and clarity from the perspective of potential users, using a predefined scoring rubric. Five criterion will be scored from 0–2, allowing a total score to summarize the overall accessibility of the data: *“Easy to find (Number of clicks from homepage)”, “Easily accessible/viewable”, “Use of graphs / visualizations”, “Main findings summarized”* and *“Glossary / guidance for interpretation”*

#### Synthesis of Results

The results of this scoping review will be presented in tables with data for each country and their respective PBCR or UO. For the indicators incidence, prevalence and survival, the number of reported items will be displayed along the horizontal axis in the table. For timeliness the date of access and the date of publication/last update will be displayed on the horizontal axis in the table. A predefined scoring guideline between 0-2 will be used to indicate how well accessibility and clarity are of the SoE. Patterns, similarities, and differences will be synthesized to summarize the current reporting practices. Based on these findings, recommendations will be provided regarding the quality, accessibility, harmonization and comparability of reporting across European cancer registries.

## References

1.	Parkin DM. The role of cancer registries in cancer control. *Int J Clin Oncol*. Apr 2008;13(2):102-11. doi:10.1007/s10147-008-0762-6

2.	ENCR. Welcome to the ENCR. European Network of Cancer Registries. Accessed 1 September 2025, <https://www.encr.eu/>

3.	Katalinic A, Hammas K, Taraszkiewicz L, et al. A Narrative Review of European Registries for Skin Cancer: Where Are We and Where Should We Be? *Cancers (Basel)*. Feb 5 2026;18(3)doi:10.3390/cancers18030524

4.	Mistry M, Parkin DM, Ahmad AS, Sasieni P. Cancer incidence in the United Kingdom: projections to the year 2030. *Br J Cancer*. Nov 22 2011;105(11):1795-803. doi:10.1038/bjc.2011.430

5.	Praagman J. *Kanker in Nederland - trends & prognoses tot en met 2032*. Netherlands Comprehensive Cancer Organisation (IKNL); 2022.

6.	Van Eycken LJ, Giannopoulos E, Tittenbrun Z, et al. Future of population-based cancer registries: A global perspective-A survey of population-based cancer registries. *Int J Cancer*. Oct 15 2025;157(8):1566-1576. doi:10.1002/ijc.35516

7.	Giusti F, Martos C, Negrao Carvalho R, Van Eycken L, Visser O, Bettio M. Quality indicators: completeness, validity and timeliness of cancer registry data contributing to the European Cancer Information System. *Front Oncol*. 2023;13:1219128. doi:10.3389/fonc.2023.1219128

8.	Bray F, Parkin DM. Evaluation of data quality in the cancer registry: principles and methods. Part I: comparability, validity and timeliness. *Eur J Cancer*. Mar 2009;45(5):747-55. doi:10.1016/j.ejca.2008.11.032

9.	Parkin DM, Bray F. Evaluation of data quality in the cancer registry: principles and methods Part II. Completeness. *Eur J Cancer*. Mar 2009;45(5):756-64. doi:10.1016/j.ejca.2008.11.033

10.	Smith-Gagen J, Cress RD, Drake CM, Felter MC, Beaumont JJ. Factors associated with time to availability for cases reported to population-based cancer registries. *Cancer Causes Control*. May 2005;16(4):449-54. doi:10.1007/s10552-004-5030-0

11.	Tricco AC, Lillie E, Zarin W, et al. PRISMA Extension for Scoping Reviews (PRISMA-ScR): Checklist and Explanation. *Ann Intern Med*. Oct 2 2018;169(7):467-473. doi:10.7326/M18-0850

12.	Peters MDJ, Godfrey C, McInerney P, et al. Best practice guidance and reporting items for the development of scoping review protocols. *JBI Evid Synth*. Apr 1 2022;20(4):953-968. doi:10.11124/JBIES-21-00242

13.	Pollock D, Evans C, Menghao Jia R, et al. "How-to": scoping review? *J Clin Epidemiol*. Dec 2024;176:111572. doi:10.1016/j.jclinepi.2024.111572

14.	Page MJ, McKenzie JE, Bossuyt PM, et al. The PRISMA 2020 statement: An updated guideline for reporting systematic reviews. *Int J Surg*. Apr 2021;88:105906. doi:10.1016/j.ijsu.2021.105906
