# Supplementary material for: Reporting of National Cancer Registry statistics in Europe: a scoping review
Source: ESMO Real World Data Digit Oncol. 2026 Sep 17;14:100752. doi: 10.1016/j.esmorw.2026.100752 (PMC13602766; doi:10.1016/j.esmorw.2026.100752)
Supplement: Supplementary file 2 [file mmc5.docx]

**Supplementary Appendix S2. The complete item sets used for data extraction.**

| **Item**: | **Comments/examples:** |
| --- | --- |
| Country |  |
| PBCR/UO name |  |
| Website link | Homepage of website |
| Website found | ENCR website or Google search |

Item set for website identification, including comments and examples for data extraction.

| **Item**: | **Comments/examples** |
| --- | --- |
| Country |  |
| Name SoE | Preferably in native language of their country |
| SoE type | Cancer report, Dashboard tool, Factsheet, Document containing tables only. |

Item set for the identification of SoEs, including comments and examples for data extraction.

| **Stratification by:** | **Comments/examples:** |
| --- | --- |
| Age | Stratified into ≥3 age groups, e.g. 0-20yr, 20-65yr, 65+yr |
| Sex |  |
| Cancer type | Stratified into ≥5 cancer sites |
| Future Trend |  |
| Stage at diagnosis | Stratified into stage I-IV or categorized into local, regional and distant metastasis. |
| Geography | E.g. incidence of different regions/provinces of country |

Item set for incidence, including comments and examples for data extraction.

| **Stratification by:** | **Comments/examples:** |
| --- | --- |
| Age | Stratified into ≥3 age groups, e.g. 0-20yr, 20-65yr, 65+yr. |
| Sex |  |
| Cancer type | Stratified into ≥5 cancer sites. |
| Stage at diagnosis | Stratified into stage I-IV or categorized into local, regional and distant metastasis. |
| Geography | E.g. prevalence of different regions/provinces of country. |

Item set for prevalence, including comments and examples for data extraction.

| **Stratification by:** | **Comments/examples:** |
| --- | --- |
| Age | Stratified into ≥3 age groups, e.g. 0-20yr, 20-65yr, 65+yr. |
| Sex |  |
| Cancer type | Stratified into ≥5 cancer sites. |
| Stage at diagnosis | Stratified into stage I-IV or categorized into local, regional and distant metastasis. |
| Geography | E.g. survival of different regions/provinces of country. |

Item set for survival, including comments and examples for data extraction.

| **Item:** | **Comments/examples:** |
| --- | --- |
| Most recent year of coverage | E.g. annual cancer report A has data from 1989-2019, note 2019. |
| Year of publication | For dashboard tools note the year of most recent update |

Item set for timeliness, including comments and examples for data extraction.

| **Criterion:** | **Statement:** | **Scoring:** |
| --- | --- | --- |
| Easy to identify | Number of clicks needed, count from homepage. | 0 = >5 clicks  1 = 3-4 clicks  2 = ≤2 clicks |
| Easy to view/use | Data published in standard format (e.g. PDF, CSV). | 0 = not in standard format available  2 = fully in standard format available |
| Use of graphs/visualization | Data is presented in graphs, besides tables only | 0 = tables only  1 = some data presented using graphs  2 = majority of data presented using graphs |
| Main findings summarized | Main findings summarized or highlighted for easier comprehension. | 0 = no summary available  2 = summary available |
| Glossary or guide for interpretation available | - | 0 = not provided  2 = fully provided guidance/glossary |

Scoring rubric for each criterion for the ranking of accessibility.
